# Aging: past, present and future

**DOI:** 10.18632/aging.100009

**Published:** 2009-01-07

**Authors:** Mikhail V. Blagosklonny, Judith Campisi, David A. Sinclair

**Affiliations:** ^1^*Ordway Research Institute, Albany, NY 12208, USA*; ^2^*Lawrence Berkeley National Laboratory, Berkeley, CA 94720, USA*; ^3^*Harvard Medical School, Boston, MA 02115, USA*

In his *Foundation* series, published in the 1950's, Isaac Asimov imagined Civilization capable of colonizing the entire Universe. This feat is unlikely to occur. Strikingly, Asimov referred to a 70-old man as an old individual who is unlikely to live much longer. Thus, in literature's most daring fantasy, the pace of aging could not be slowed. Yet, given the present pace of discovery in the aging field, this feat might become a reality within our life time, with science surpassing science fiction.

## Past

Once August Weismann had divided life into a perishable soma and immortal germ line, the soma began to be viewed as disposable. As Weismann wrote in 1889, "the perishable and vulnerable nature of the soma was the reason why nature made no effort to endow this part of the individual with a life of unlimited length" (see [1]). Weismann speculated that somatic mortality might give the individual benefits early in life, such as "a better performance of their special physiological tasks" … or "an additional material and energy available for the reproductive cells". This quote implies two hypotheses. Each of them initiated a separate direction of thought in gerontology. First, mortality can result from benefits at young ages (e.g., better performance). This idea is the root of the antagonistic pleiotropy theory later developed by Medawar (1952) [2] and Williams (1957) [3]. Second, mortality can result from allocation of limited resources for reproduction. This is the root of the allocation of resources or disposable soma theory developed by Kirkwood [4]. The allocation hypothesis predict that the fewer the resources for reproduction, the shorter lifespan.

Experimental data were available to distinguish two hypotheses as early as 1917. It was shown that caloric restriction (CR) - a reduction in food intake without malnutrition - extends life span and prevents age-related infertility in rodents [5]. These data were initially forgotten, but have now been reproduced numerous times. In 1930's, McCay and his colleagues also found that CR prolongs life span in rodents. The significance of CR is fully appreciated only now, knowing that nutrients modulate cellular signal transduction pathways that include AMPK, Sirtuins and TOR. But for a long time the effect of CR on longevity remained just a phenomenon, albeit an important one. Aging remained an unsolved mystery.

In 1950's, aging began to be understood from an evolutionary perspective. Because organisms tend to die from external causes in the wild, the probability of survival to old age is low. Therefore, the force of natural selection weakens with age. Natural selection, which is strong early in life, can favor antagonistically pleiotropic genes (AP genes), genes that provide benefits early in life but are harmful later. By 1957, it was generally accepted that there were genes that are beneficial early in life, but cause aging phenotypes at older ages. This idea predicts that the inactivation of some genes will extend life span, but at the cost of development or reproduction. The identity of such genes remained enigmatic for two reasons. First, there were no technologies to screen for such genes at that time. Second, it was expected that these genes must differ among species. For example, in mammals there might be genes responsible for rapid calcification of bones that lead to late-life calcification of atherosclerotic plaques (an example suggested by Williams).

Nematodes such as *Caenorhabditis elegans* do not have bones or a circulatory system that is susceptible to atherosclerosis. So, aging of *C. elegans* and other simple organisms was expected to be irrelevant to human aging. Because *C. elegans* has a short (~3 weeks) life span and is more suitable for genetic screens than monkeys, or, needless to say, humans, antagonistically pleiotropic genes remained hypothetical.

Instead, research efforts were focused on obtaining evidence for the allocation of resources hypothesis. Aging was assumed to result from the random accumulation of damage, resulting in chaos and an increase of entropy that could not be regulated or prevented. In 2006, some investigators declared that the problem of aging had been solved [6,7]. This view declared that aging is just deterioration and functional decline due to an accumulation of random molecular damage from myriad causes, and refractory to substantive intervention [6,7].

But what about antagonistically pleiotropic genes predicted in 1950's? It was pointed out that "one of the problems with this view, though, is that there are, in fact, very few clear-cut examples of candidate pleiotropic genes other than p53" [4].

## Present

The first successful screens for genes that postponed aging began in the mid 1980's. Despite common opinion that genes that control aging were unlikely to exist, Klass performed a mutagenesis screen for long-lived *C. elegans* mutants and found candidates [8], one of which, age-1, was characterized by Johnson and colleagues [9]. In 1993, Kenyon and colleagues, also screening for long-lived C. elegans, found that mutations in the gene daf-2 increases the longevity of *C. elegans* hermaphrodites by more than two-fold compared to wild type nematodes [10]. Daf-2 was already known to regulate formation of the dauer state, a developmentally arrested larval form that is induced by crowding and starvation. Kenyon et al. suggested that the longevity of the dauer results from a regulated life span extension mechanism. This discovery provided entry points into understanding how life span can be extended [10]. So Age-1 and Daf-2 appeared to be antagonistically pleiotropic (AP) genes. As predicted by the AP theory, Age-1 or Daf-2 inactivation extends life span, but at the cost of impaired development or fertility [9-10]. Subsequent studies showed that Age-1 and Daf-2, as well as Daf-16 and Daf-18 (mutations in which suppress life span extensions caused by Daf-2 and Age-1 mutations) act in the same signal transduction pathway [11]. Similar genes were soon identified in yeast, *Drosophila* and mammals, thereby establishing the existence of universal genes that control aging [12]. Daf-2 encodes an insulin receptor family member [13], and Age-1 encodes a homologue of mammalian PI-3K catalytic subunits [14], which transduce the receptor-mediated signals. On the other hand, Daf-18 is homologue of the tumor suppressor PTEN [12], which suppress Daf-2 and Age-1 signals.

Soon after these discoveries, the yeast chromatin-associated protein Sir2 (silent information regulator-2) and its mammalian ortholog SIRT1, as well as other sirtuins, were found to regulate longevity in diverse species, ranging from yeast to mammals, stemming from fundamental discoveries in Lenny Guarente's lab. Sirtuins are also involved in nutrient sensing (and are important mediators of the effects of CR), DNA damage sensing and responses, genomic stability, metabolic diseases, and cancer in mammals [15-24]. Some sirtuins seem to act in the same nutrient-sensing longevity pathway that is regulated by TOR (target of rapamycin) [25]. The sirtuin/TOR pathway extends life span by mechanism that overlap with caloric restriction (CR) [25]. Accordingly, in 2003, it was demonstrated that knocking down TOR in *C. elegans* more than doubles the life span [26]. Likewise, in *Drosophila*, genetic inhibition of the TOR pathway both upstream and downstream of TOR extends life span [27]. Similarly, inhibition of TORC1 signaling in yeast extends life span [28,29].

Thus, genetic studies have now firmly established that aging is regulated by specific genes conserved from yeast to mice [23,30-34].

Independently of discoveries of AP genes in model organisms, research in cellular senescence yielded complementary results. Cellular senescence is now widely recognized as an essential tumor suppressor mechanisms, and is not a decline due to cellular degeneration. In contrast, the senescence response is an active process caused by activation of both DNA-damage responses (DDR) and mitogen-activated pathways. Senescent fibroblasts acquire phenotypes that include hyper-secretion of inflammatory cytokines and other tissue-altering molecules and hypertrophic morphologies [35-42]. Senescent cells can alter the behavior of neighboring cells [35], and may drive aging and age-related diseases [43]. Cellular senescence can be prevented by knocking out cell cycle inhibitors such as p53, pRb, p21 and p16 [44-48]. This disabling of cell cycle brakes (and thus immortalization) can occur as a consequence of molecular damage and subsequent mutation. Normally, the DNA damage response inhibits cell cycle progression. Consistent with cellular senescence being tumor suppressive, activation of growth-promoting pathways such as Ras, Raf, MEK and PI-3K leads to senescence [49-51]. Inhibition of mTOR decelerates cellular senescence [42].

Finally, the same signaling pathways (for example, the PI-3K pathway) that are involved in cancer are also involved in cellular and organismal aging. Therefore, progress in cancer research fosters progress in aging research [52-57]. Certain ‘anticancer drugs' are potential ‘anti-aging drugs', and -- vice versa -- inhibition of aging pathways are a strategy to prevent cancer [58].

The discovery of telomerase in 1987 [59] lead to an understanding of how telomere attrition causes a DNA damage response (DDR), culminating in cell cycle arrest and cellular senescence [46, 60-62]. Recent findings show that expression of telomerase can extend lifespan in mice, providing the mice carry extra copies of the p53, p16 and Arf tumor suppressor genes, which renders them cancer-resistant [63].

Thus, aging is regulated by signaling networks encompassing nutrient-sensing, and mitogen-activated, stress-responsive and DDR signaling pathways. Furthermore, CR modulates portions of the same signaling networks. These networks were conserved during evolution and includes genes that are antagonistically pleiotropic: they drive growth and development early in life and aging later in life.

## Future

Of great interest and excitement, aging now appears to be regulated at least in part by signal-transduction pathways that can be manipulated pharmacologically. Prototype anti-aging drugs are available now to treat age-related diseases, and are predicted to slow aging processes. Modulators of sirtuins have been discovered [64] that mimic CR and mitigate certain age-related diseases [65-67]. The TOR pathway is another target. Ironically, TOR itself was discovered as target of rapamycin in yeast [68] (Sirolimus or Rapamune), a clinically available drug that is tolerated even when taken in high doses for several years. Rapamycin has potential as a therapy for most of not all age-related diseases [69-71], and metformin, an anti-diabetic drug and activator of AMPK, which acts in the TOR pathway retards aging and extends lifespan in mice [72].

Thus, recent paradigm shifts in aging research has put signaling pathways (growth-promoting pathways, DNA damage responses, sirtuins) at front stage, has established that aging can be regulated and can be inhibited pharmacologically.

At this opportune time, *Aging*(Impact Journal on Aging or Impact Aging) is launched. This journal embraces the new gerontology. Recent breakthrough in gerontology is due to an integration of different disciplines, such as genetics and development in model organisms, signal transduction and cell cycle control, cancer cell biology and DNA damage responses, pharmacology, and the pathogenesis of many age-related diseases. The journal will focus on signal transduction pathways (IGF- and insulin-activated, mitogen-activated and stress-activated pathways, DNA-damage response, FOXO, Sirtuins, PI-3K, AMPK, mTOR) in health and disease. Topics include cellular and molecular biology, cell metabolism, cellular senescence, autophagy, oncogenes and tumor suppressor genes, carcinogenesis, stem cells, pharmacology and anti-aging agents, animal models, and of course age-related diseases such as cancer, Parkinson's disease, type II diabetes, atherosclerosis, macular-degeneration, which are deadly manifestations of aging. The journal will also embrace articles that address both the possibilities and the limits of the new science of aging. Of course, the possibility that the diseases of aging can be delayed or treated by drugs that affect the overall aging process, thus potentially extending healthy lifespan, is long-standing dream of mankind.
